# Additive Diversity Partitioning of Fish in a Caribbean Coral Reef Undergoing Shift Transition

**DOI:** 10.1371/journal.pone.0065665

**Published:** 2013-06-11

**Authors:** Gilberto Acosta-González, Fabián A. Rodríguez-Zaragoza, Roberto C. Hernández-Landa, Jesús E. Arias-González

**Affiliations:** 1 Laboratorio de Ecología de Ecosistemas de Arrecifes Coralinos, Centro de Investigación y de Estudios Avanzados del I.P.N-Unidad Mérida, Mérida, Yucatán, México; 2 Laboratorio de Ecosistemas Marinos y Acuicultura (LEMA), Departamento de Ecología, CUCBA, Universidad de Guadalajara, Jalisco, México; National Institute of Water & Atmospheric Research, New Zealand

## Abstract

Shift transitions in dominance on coral reefs from hard coral cover to fleshy macroalgae are having negative effects on Caribbean coral reef communities. Data on spatiotemporal changes in biodiversity during these modifications are important for decision support for coral reef biodiversity protection. The main objective of this study is to detect the spatiotemporal patterns of coral reef fish diversity during this transition using additive diversity-partitioning analysis. We examined α, β and γ fish diversity from 2000 to 2010, during which time a shift transition occurred at Mahahual Reef, located in Quintana Roo, Mexico. Data on coral reef fish and benthic communities were obtained from 12 transects per geomorphological unit (GU) in two GUs (reef slope and terrace) over six years (2000, 2005, 2006, 2007, 2008, 2010). Spatial analysis within and between the GUs indicated that the γ-diversity was primarily related to higher β-diversity. Throughout the six study years, there were losses of α, β and γ-diversity associated spatially with the shallow (reef slope) and deeper (reef terrace) GUs and temporally with the transition in cover from mound corals to fleshy macroalgae and boulder corals. Despite a drastic reduction in the number of species over time, β-diversity continues to be the highest component of γ-diversity. The shift transition had a negative effect on α, β and γ-diversity, primarily by impacting rare species, leading a group of small and less vulnerable fish species to become common and an important group of rare species to become locally extinct. The maintenance of fish heterogeneity (β-diversity) over time may imply the abetment of vulnerability in the face of local and global changes.

## Introduction

One of the central topics in conservation is the analysis of species diversity patterns and composition for the purpose of differentiating, characterizing and preserving natural communities [1], [2]. Fish species diversity patterns may differ and be influenced by different “driving forces” at different spatial and temporal scales [2]. In coral reefs, at both local and regional scales, changes in habitat structure [3]–[5], habitat area (species-area relationship) [6]–[9] and the presence or absence of certain key species or functional groups [1], [10]–[12] may have a direct and immediate impact on coral reef fish diversity [13], [14].

One way of assessing variation in diversity is through the additive partitioning of diversity [2], [4], [15]–[19]. Additive partitioning estimates the relative contributions of α-diversity, which is represented by the average number of species at a certain scale, and β-diversity, which is the average turnover or change in the composition and abundance of species between two analyzed scales, in relation to total diversity [2], [20]–[22].

This analysis has recently been used in Caribbean and Pacific coral reefs to perform additive partitioning for fish diversity in hierarchical scaled studies and to analyze the variables that modulate the β-diversity of coral reef benthic communities [4], [5], [17], [19]. Additive partitions have also been used to analyze hierarchical patterns of species diversity across landscapes and regions [2] and to assess multiple spatial scales [23] to study the effects of landscape connectivity [24], paleo-biodiversity patterns [25] or spatiotemporal patterns [26]–[29]. To date, this method has not been applied to understanding the relationship between the spatial variation in coral reef fish diversity and benthic shift transitions such as that observed in the Caribbean Sea.

In the last four decades, many coral reefs in the Caribbean Sea have been affected by a shift in benthic coral reef communities from a dominance of hard coral cover to one of fleshy macroalgae [30]–[35]. This shift transition may result in habitat and rugosity homogenization, particularly represented by a loss of cover or the erosion of mound corals such as the *Montastraea* complex [36]. This may drive spatiotemporal changes in coral reef fish species composition, as it has been observed in multiple studies that fish diversity is strongly related to coral cover [5], [10], [11], [37] and habitat complexity [7], [11], [38]–[40]. Although multiple studies have focused on the causal forces that produce a shift transition (e.g. [35]), little is known about how the additive components (α- and β-diversity) of total diversity (γ) may be affected by this global event in coral reefs.

Coral reefs are spatially structured across a depth gradient from the coast to the open ocean. In the Caribbean, the reef lagoon, crest and front are the systems that receive direct impacts from coastal development and tourism. Deep geomorphological structures may also be considerably affected, but as these structures are farther from the coast (600–1000 m) and in deeper water (>20 m), they may be more resistant to climate change and coastal development. A pioneering study of shifts in the benthic structure of the reef fronts of eleven coral reefs in Quintana Roo, Mexico demonstrated that coral reefs associated with intensive coastal tourism development were largely dominated by fleshy macroalgae (82%) and that the coral cover (13%) was poor, whereas reefs with few coastal tourism activities exhibited a more balanced coverage of fleshy macroalgae and corals (38% and 22% respectively) [41]. In our case, we found at the beginning of our investigation (in the year 2000) that shallow geomorphological units of our studied reef, the reef slope (ca. 15 m), had a balanced coverage of coral and fleshy macroalgae, whereas the deepest geomorphological unit, the terrace (ca. 20 m), had a higher coverage of coral: the reef was undergoing a shift transition. Therefore, an additional issue involved in a temporal shift transition of additive partitioning of diversity is how α- and β-diversity change in space and across reef geomorphology during the shift transition event. Ecological studies of additive partitioning indicate that β-diversity is the most significant contributor to γ-diversity (see [25] for a review). Belmaker et al. [17], Rodríguez-Zaragoza and Arias-González [4] and Rodríguez-Zaragoza et al. [19] recently proposed coral colonies, coral cover, topographic complexity and shelter availability as the main components driving fish β-diversity change. A question proposed by Sepkoski [42] concerning the drivers of total diversity partitioning events such as regime shifts in coral reefs is as follows: to what extent do total changes in diversity (γ-diversity) reflect community-level changes (α-diversity) and between-community spatial and temporal changes (β-diversity)?

In this paper, our primary objective was to explore patterns in coral reef fish diversity across two different spatial (transects and geomorphological units) and temporal (years) scales using the additive components of total diversity. In particular, we hypothesized that this shift would decrease fish γ-, β- and α-diversity, although it is expected that in a threatened or collapsed system, α-diversity would increase at the expense of a proportional amount of β-diversity. Our second objective was to quantify the additive diversity partitions within two geomorphological units (reef slope and terrace) in each study year and assess the main coral reef benthic drivers associated with α- and β-diversity change. Although the loss of fish diversity with loss of coral cover has been widely documented in coral reef ecology [43], the consequences for α-diversity and β-diversity as components of γ-fish diversity through space and time are largely unknown. This information is useful for the management and conservation of coral reef fish diversity.

## Methods

### Study Area

The coral reef system of Mahahual is located in the northern part of the Mesoamerican Barrier Reef System in the state of Quintana Roo, within the touristic area known as Costa Maya (18°42′50.53″N; 87°42′13.53″W) (Fig. l). In 2000, Mahahual’s reef was listed as one of the better reefs, in terms of its condition, in the Mexican Caribbean [44]. This reef is characterized by a well-defined zonation with five geomorphological units: lagoon (L, 3 m depth); crest (C, emergent); front (F, 5 m depth); slope (S, 12 m depth); and terrace (T, 18 m depth) [45]. In 2000–2001, a pier was constructed to receive tourist cruise ships in the northern part of the reef. This encouraged the creation of associated urban and touristic infrastructure, including restaurants, artificial beaches, navigation channels and hotels. These developments together with hurricanes, bleaching events and over-fishing have contributed to a generalized phase shift in all of the studied geomorphological units.

### Field and Laboratory Work

**Figure 1:**
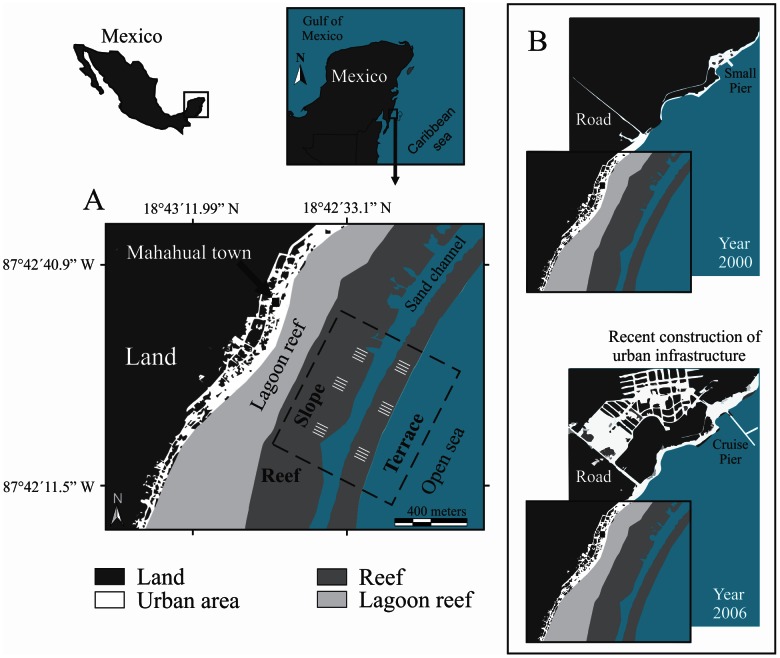
Locations of study sites at Mahahual Reef. Sampling sites with their respective repetitions (A) and changes in the use of the coast between the years 2000 and 2006 (B).

Data from the coral reef fish and benthic communities were obtained in November in the years 2000, 2005, 2006, 2007, 2008 and 2010. A balanced design with two spatial scales (transects: 12 per geomorphological unit (GU) and GUs: reef slope+terrace) and a one-decade time scale (from 2000 to 2010) was used. Fish and benthos were censused using 50-m-long and 2-m-wide transects positioned at depths where the largest coral reef *Montastraea* system was located: the reef slope (ca. 12 m depth) and terrace (ca. 18 m depth). Within each geomorphological unit, four sampling transects were selected at three different fixed sites that were separated from one another by 250–300 m (Fig. 1). At each site, fish >15 cm were visually censused by SCUBA diving. First, four transect lines separated by 50 m were positioned across each fixed sampling site, and then, two censuses were performed, the first recording reef fishes and the second video recording the benthic communities. Two experienced observers, one for the years 2000, 2005, 2006 and 2007 and the other for 2008 and 2010 conducted visual censuses for all years of sampling. Using the same sampling protocol and a standardized record of species richness, abundance and sizes minimized the bias between observers. We recorded only the species that corresponded to pelagic, demersal and benthic fish, as those are the most conspicuous species that determine the “visible” fish assemblage structure [38], [45]. We did not include cryptic fish species, as they take too long to count accurately within a transect. The difficulty of visually detecting small-bodied fish is well known and may produce underestimates in the abundance of small cryptic fish such as Gobiidae, Apogonidae, and Blenniidae [46].

The benthic community was surveyed by recording it using an underwater video camera at a distance of 40 cm above the substrate along each 50 m transect [37]. The camera was held approximately 50 cm to the side of the tape and approximately perpendicular to the substrate. Each videotransect was then sampled using 40 frames and 13 systematically dispersed points: 520 points per transect. The benthos under each sampled point was identified to the lowest taxonomic group and life form possible. For several analyses, data were grouped into the following major benthic categories: scleractinian corals, octocorals, hydrocorals, articulated calcareous algae, encrusting calcareous algae, fleshy macroalgae, turf, sponge, sand, calcareous substrate, and rubble. Scleractinian corals were grouped into the following coral categories [47]: mound, boulder, encrusting, finger, branching, tabular, fleshy and leaf. Permission to conduct field research at the Mahahual reef was not necessary because this area does not have any conservation status. The study was conducted on free-living wild animals in their natural habitat and solely involved observations and video recordings of animals and noninvasive measurements.

### Data Analysis

#### Rarefaction curves

The number of species and optimum sampling effort were evaluated based on observed (Mao Tau function) and expected species (bootstrap) using transect-based rarefaction curves [19]. The bootstrap method is based on the proportion of transects containing each observed species [48]. The total species richness was compared among years per geomorphological unit and for the overall reef with individual-based rarefaction curves, using upper and lower 95% confidence intervals [49]. Both kinds of rarefaction curves were constructed with 10,000 randomizations without replacement, using EstimateS V8.2 [50]. Chi-square (*χ* ^2^) goodness-of-fit [51] was used to contrast the observed and expected species richness per geomorphological unit and for the reef. We estimated species’ rarity based on abundance and incidence as a way of assessing the spatial-temporal loss of β-diversity; abundance was evaluated based on the numbers of singletons (species represented by a single individual) and doubletons (species with only two individuals) among transects, and incidence was characterized by the numbers of unique (species recorded in a single transect) and duplicate (species recorded in only two transects) species [22]. Finally, the unshared species were estimated for each geomorphological unit and for the whole reef across the studied years.

#### Diversity additive partitioning

We measured α-diversity and β-diversity as components of γ-diversity among subdivisions of fish assemblages on the basis of transects, geomorphological units and years. The proportion of total diversity within subdivisions in a given dimension (i.e. space and time) provides a natural measure of similarity among the subdivisions [26], [52], [53]. The additive partitioning model was used to evaluate the contribution of α-diversity at the transect scale (α_transects_) within the reef slope, the reef terrace, and the reef as a whole (combining both reef slope and terrace). β-diversity was evaluated at two spatial scales (β_transects_ and β_gemorphological units (GUs)_) within the reef slope and reef terrace and for the entire reef (reef slope+reef terrace).

Diversity partitioning is an analytical procedure in which a measure of total species diversity is decomposed at spatial or temporal levels in a hierarchical sampling design [54]. For spatially structured sampling designs, the total species diversity at the conclusion of an inventory can be broken down into α- and β-components such that α-diversity corresponds to the average diversity within a sampling unit (i.e., transects in our case) and measures of β-diversity correspond to average diversity among sampling units [55]; in our case, either among transects or between geomorphological units. Therefore our design was γ-diversity  =  α ¯_transects_+β_transects_+β_GU_. We estimated α- and β-diversity using PARTITION v3 [56]. This program tests the statistical significance of α- and β-diversity by spatial scale in terms of null models. The additive partitions were constructed based on individuals, with unweighted transects in a balanced design. Unrestricted, individual-based randomization was used. This approach is one of the simplest null models and uses complete randomization, where individuals (fish) were randomized among all transects across geomorphological units and years. In other words, the species abundances from all samples at a given level were combined to create a single species-abundance distribution [57], [58]. This distribution was used to randomly assign individuals (without replacement) to samples, such that the initial size (number of individuals) of the sample was maintained. As with the observed data, the randomized data were partitioned and the diversity estimates were obtained. Null models were constructed with 10,000 randomizations per spatial level to estimate p-values between observed and expected diversity [59]. Statistical significance was assessed based on the proportion of null values that were greater than (or less than) the observed α- and β-diversity. Deviations from this null model indicated a non-random spatial distribution of individuals [17].

#### Statistical analysis

The β-diversity within each geomorphological unit and within the reef was measured by pairwise comparisons among all transects, using presence-absence data. We used Lande’s β-diversity (β = S−α ¯), which was estimated with the equation β = (b+c)/2, where b is the total number of species that occur in the neighboring transect but not in the focal one and c is the total number of species that occur in the focal transect but not in the neighboring one [60]. This means that the b component measures species gain and the c component species loss relative to the focal transect [60]. We used the Vegan package [61] in the R statistical software program [62] to estimate β-diversity for the presence-absence data.

Due to the lack of independence among years (time scale), a two-way crossed ANOVA with a mixed-effects model (type model III) was used to contrast the -diversity (average species richness per transect) and β-diversity for presence-absence data within geomorphological units and within each year [50]. This design considered the spatial scale (geomorphological units) as a fixed factor and the time scale (years) as a random factor. Additionally, a one-way ANOVA based on random effects (model II) was used to compare the β-diversity of the reef as a whole (reef slope+reef terrace) among years [51], [62]. The β-diversity within years was transformed using a Box-Cox transformation (X^0.82^) to meet statistical assumptions. We tested the statistical assumptions with a Shapiro-Wilk normality test and Levene equal variance test [62].

An additional method was applied to evaluate fish assemblage changes between the reef slope and terrace over the years. A two-way crossed permutational multivariate analysis of variance (PERMANOVA), based on a mixed-effects model following the two-way ANOVA design, was used to assess the spatiotemporal variation in species composition and abundance. This particular design included/permitted the following: i) the geomorphological unit×year interaction, which tested the generality of species turnover of geomorphological units over years; ii) the year factor, which tested the temporal variability among the six studied years; and iii) the geomorphological unit factor, which tested the consistency of species turnover between geomorphological units, above and along with the potential variability in its change among years. The PERMANOVA was performed using a Bray-Curtis similarity matrix built with previously fourth-root-transformed data. The statistical significance of the PERMANOVA was tested with 10,000 residual permutations under a reduced model and type II (conditional) sums of squares [63]. The dissimilarity contribution of the species between geomorphological units through years was estimated using a two-way similarity percentage analysis (SIMPER). Multidimensional analyses were conducted in Primer V6 & PERMANOVA+ [63]–[65].

Canonical redundancy analysis (RDA) was used to visualize and describe the relationships among the components of fish α ¯_transects_, β_transects_ and γ-diversity with coral reef benthic community for each geomorphological unit in each year. Redundancy analysis was performed using the CANOCO v4.5 Software [66]. Multicollinearity was evaluated among environmental variables because the RDA model output could be modified. Only environmental variables with Pearson correlation (r) values below 0.90 were selected [67], [68]. However, RDA analysis was performed using a variance inflation factor (VIF) below 10 to avoid severe multicollinearity [69]. However, Graham [67] suggested that VIF values as low as two may have significant impacts. The association of the dataset was validated using 9,999 permutations under a reduced model [70].

## Results

A total of 11,134 individuals were recorded from 33 families, 58 genera and 118 species. The families Serranidae, Pomacentridae, Haemulidae, Labridae, Lutjanidae, and Scaridae accounted for 54% of the total species richness. The observed and expected transect-based rarefaction curves indicated that the sampling effort for geomorphological units and for the overall reef was representative of species richness. The observed species curves obtained using the bootstrap exhibited no differences at the geomorphological unit or reef levels between 2000, 2005, 2006, 2007, 2008 and 2010 (Chi-square test; *p*<0.05).

The individual-based rarefaction curves indicated a decrease in the total number of species at the reef level and in the reef terrace GU between 2000 and subsequent years. The decline in species was most obvious between the year 2000 and 2005, 2008 and 2010. In 2000, the overall reef and the reef terrace GU presented the highest total species richness, whereas during and after 2005, the total species richness strongly decreased. The main significant differences and decreases were observed in the years 2005, 2008, and 2010 (see Fig. S1).

**Figure 2:**
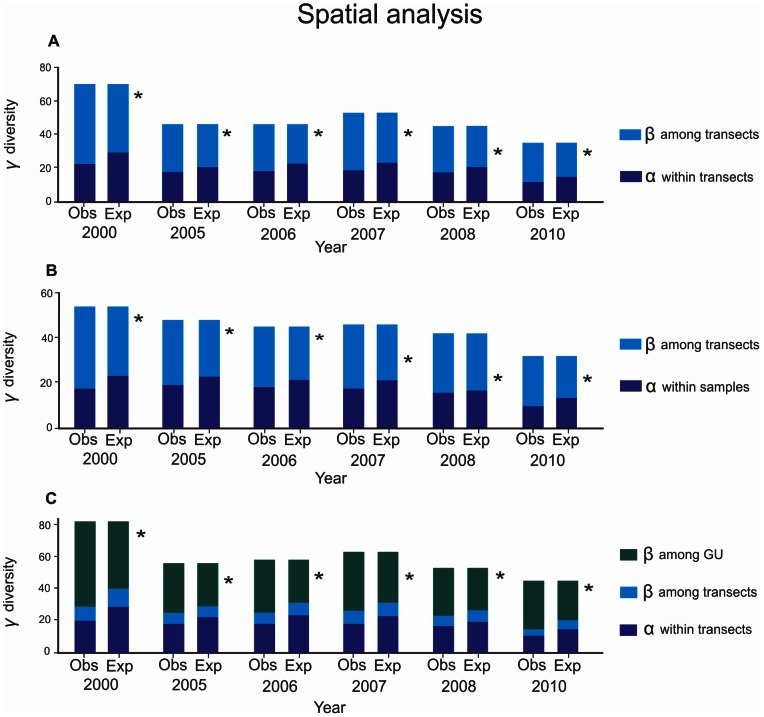
Additive partitioning of reef fish diversity. Spatial analysis: reef terrace (A), reef slope (B), and whole reef (C). The levels of significance between observed (Obs) and expected (Exp) values are shown to the right of the graph: * = p<0.0001. Geomorphological units = GU.

The partitioning of fish species diversity within each geomorphological unit showed that the β_transects_ diversity within each GU was the only additive component that showed clear differences between observed and expected - and β-diversities. β_transects_ was significantly higher than expected by chance (Figs. 2A and 2B). This suggests that β_transects_ was a real biological component influenced by different habitat structure variables and not by random patterns. In 2000, α ¯_transects_ contributed from 18 to 22 species and β_transects_ contributed 36 to 48 species. In 2005–2007, α ¯_transects_ contributed with 18 to 19 species and β_transects_ contributed from 27 to 34. Finally in 2008 and 2010, α ¯_transects_ contributed 10 to 18 species and β_transects_ contributed from 22 to 28 species (Figs. 2A and 2B).

The whole reef partitioning analyses indicated that α ¯_transects_ and β_transects_ did not exhibit significant differences between observed and expected estimates, but β_GU_ was significantly higher than expected by chance (Fig. 2C). In 2000, α ¯_transects_ contributed 20 species, β_transects_ 9 species, and β_GU_ 53 species. In contrast, during 2005–2007, α ¯_transects_ contributed 18 species, β_transects_ 7 to 8 species, and β_GU_ from 30 to 37 species. Finally, in 2008 and 2010, α ¯_transects_ contributed 11 to 17 species, β_transects_ 4 to 7 species and β_GU_ only 30 species (Fig. 2C).

**Figure 3:**
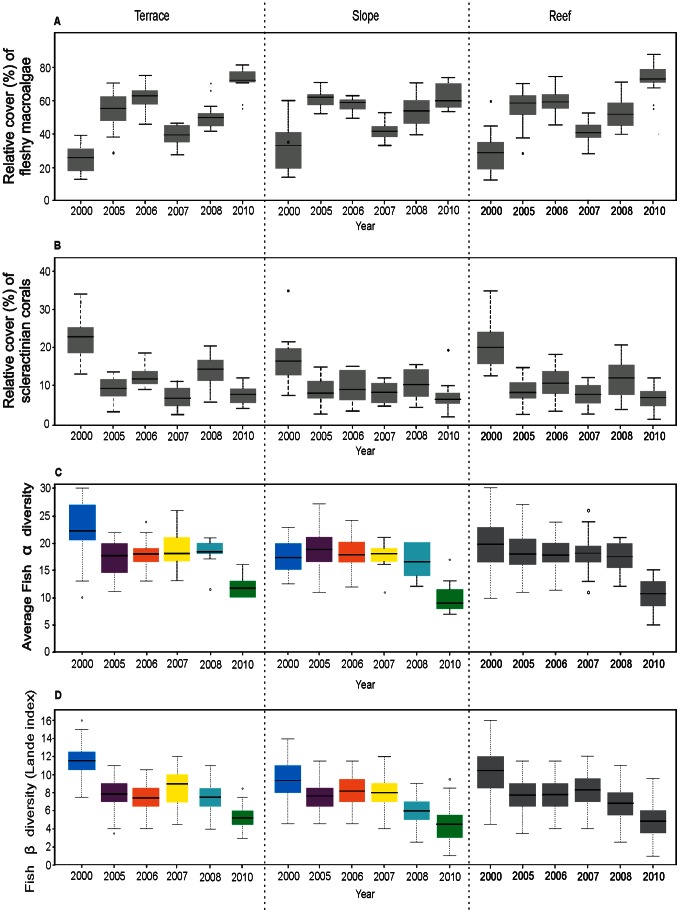
Relative cover of scleractinian corals and fleshy macroalgae (A), fish α ¯-diversity (B), and fish β-diversity (C). The horizontal line in the box plot indicates the mean and the box indicates the upper and lower quartiles, with the vertical lines representing the minimum and maximum values.

The results of the two-way crossed ANOVA revealed that the -diversity was not significantly different between geomorphological units but that it was significantly different among years (Table 1). The interaction between the year and geomorphological unit factors was not significant (Table 1, Fig. 3C). Therefore, -diversity gradually decreased over time. The two-way crossed ANOVA for β-diversity also demonstrated that β-diversity was only significantly different among years and in terms of the interaction of year and geomorphological unit (Table 1). This means that β-diversity varied between geomorphological units over the years, decreasing from 2000 to 2010 (Fig. 3D). The highest β-diversity was recorded in the year 2000, when the shift transition was relatively weak. In contrast, the lowest species turnover was estimated in the years 2008 and 2010, when the shift transition was stronger (Fig. 3D).

**Table 1:** Results of two-way crossed and mixed-effect ANOVAs and the one-way random-effect ANOVA.

Two-way mixed-effect ANOVA				
α ¯ diversity (mean species richness) per GU				
Source	df	F-ratio		P-value
Year	5	9.670		**0.0131**
GU	1	2.538		0.1720
Year×GU	5	2.144		0.0640
Residual	132			
***β*** ** diversity among UG and among Years**				
**Source**	**df**		**F-ratio**	**P-value**
Year	5		16.039	**0.0042**
GU	1		3.826	0.1078
Year×GU	5		10.058	**<0.0001**
Residual	780			
**One-way** random-effect **ANOVA**				
Reef's α ¯ diversity among years				
**Source**	**df**		**F-ratio**	**P-value**
Years	5		22.278	**<0.0001**
Residual	138			
**Reef's ** ***β*** ** diversity among years**				
**Source**	**df**		**F-ratio**	**P-value**
Years	5		146.933	**<0.0001**
Residual	786			

Geomorphological units: GU.

The one-way random-effect ANOVA indicated that α ¯-diversity and β-diversity were significantly different among years at the whole reef level (Table 1), showing a decline in average species richness and turnover throughout the analyzed period (Figs. 3C and 3D). We found that a large number of unshared species were lost (18 species, 22% of total species) from the year 2000 to 2010 (see Table S1). During this period, species rarity decreased at both geomorphological units across the studied years. Singletons and unique species showed the most important changes for both the reef slope and terrace, whereas doubletons and duplicated species only changed in the reef terrace. The results for the reef level were complementary (similar patterns) to those obtained for the reef slope and reef terrace. (see Fig. S2 and Table S1).

The two-way crossed PERMANOVA showed that species composition and abundance were statistically different between geomorphological units and among years as well as in terms of the interaction between these factors (Table 2). The differences were produced by a large change between the geomorphological units across the studied years. The species that contributed most to the differences in composition and abundance for each geomorphological unit and year are shown in the supplementary electronic material (Table S2).

**Table 2:** Results of two-way crossed and mixed-effect PERMANOVA based on Bray-Curtis similarity matrix.

Source	df	Pseudo-F	P(perm)	Unique perms
Year	5	10.614	**0.0001**	9852
GU	1	4.445	**0.0125**	9404
Year×GU	5	2.353	**0.0001**	9829
Residual	132			

PERMANOVA tested the temporal and spatial variation of fish species composition and abundance between geomorphological units (GU) across years (from 2000 to 2010).

**Figure 4:**
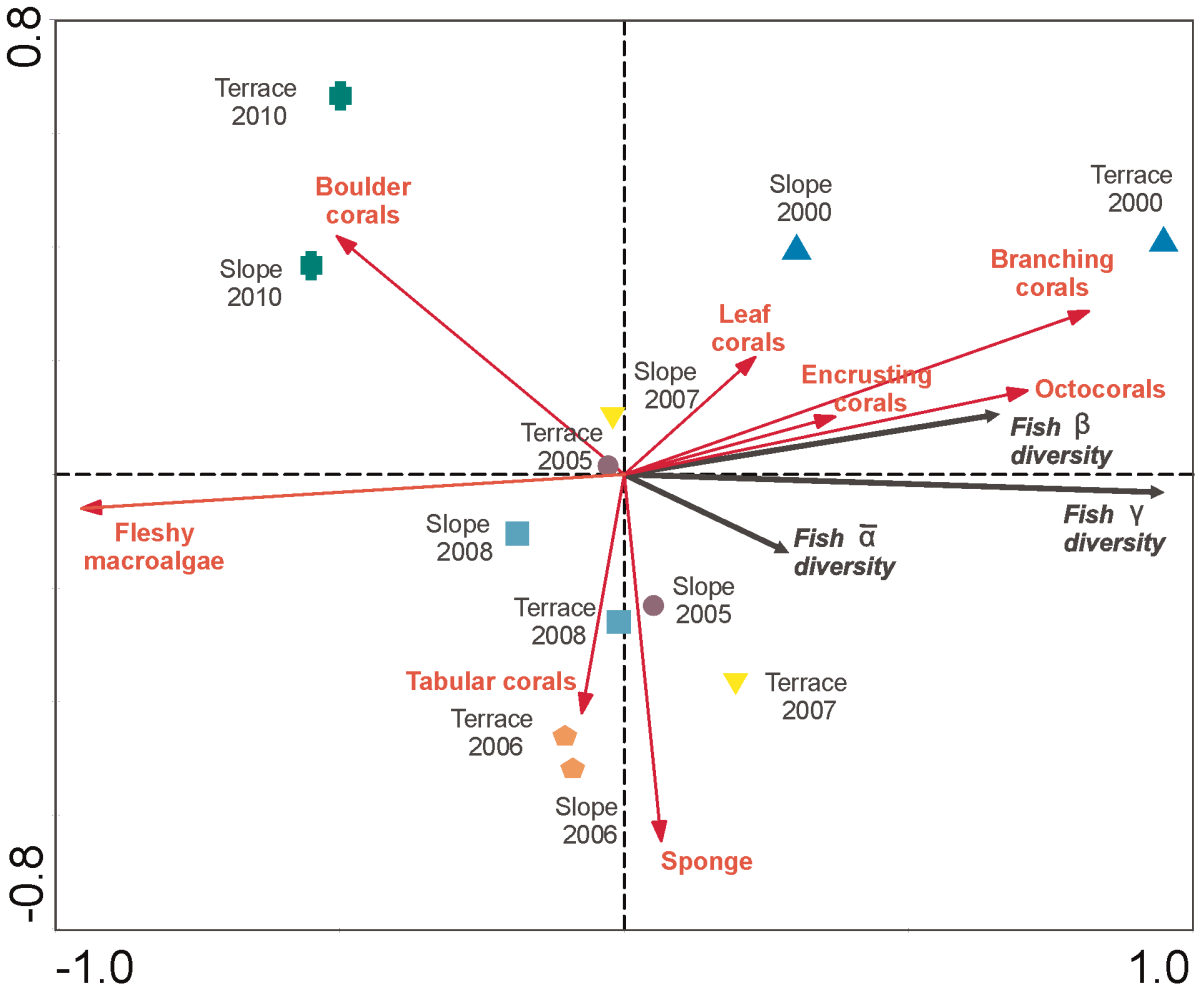
Canonical redundancy analysis triplot for fish α ¯, β, and γ diversity between the geomorphological units across the studied years. Test of significance of all canonical axes: Trace = 0.983, p = 0.0077. Environment variables were selected through stepwise forward-selection procedure: fleshy macroalgae (λ = 0.88, p<0.001), branching corals (λ = 0.05, p = 0.012), sponges (λ = 0.01, p = 0.129), boulder corals (λ = 0.01, p = 0.064), encrusting corals (λ = 0.01, p = 0.332), leaf corals (λ = 0.01, p = 0.179), tabular corals (λ = 0.01, p = 0.249), octocorals (λ = 0.01, p = 0.216).

The RDA triplot presented a good fit and high statistical significance (Trace = 0.983, *p = *0.0077), where the geomorphological units were observed to separate according to year sampled (Fig. 4). Fish α ¯_transects_, β_transects_ and γ-diversity showed greater contributions to the geomorphological units in the year 2000 and reduced contributions from 2005 to 2010. The variables that explained these patterns were the percent cover of fleshy macroalgae, branching corals (*Acropora cervicornis*), octocorals, sponges, boulder corals (*Siderastrea* spp., *Porites astreoides,* and *Diploria* spp.), tabular corals (*Acropora palmata*), encrusting corals (*Agaricia* spp.) and leaf corals (*Agaricia tenuifolia*).

**Figure 5:**
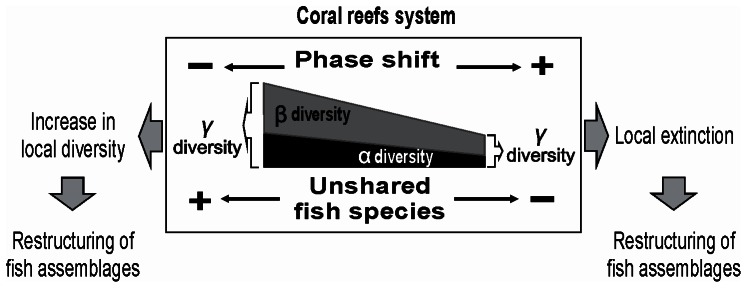
Conceptual model of the phase shift’s effect on reef fish diversity. Change in the proportions of α- and β- diversity with regard to the total fish diversity (γ) and the loss of unshared species in relation to the degree of phase shift (see electronic supplementary information, Table S1), implying a local extinction of species and change in the fish assemblage structure.

In the RDA forward selection, fleshy macroalgae were found to make an important contribution to the model, although this benthic categories was highly collinear with mound corals (*i.e., Montastraea* complex) in a negative linear relationship, and hence, they were not included in the analysis. Fleshy macroalgae presented an important negative correlation with α ¯_transects_, β_transects_ and γ-diversity, and they increased at the geomorphological unit level from the years 2005 to 2010 (also see Fig. 3A). Similarly, boulder corals exhibited an important cover increase in 2010. In contrast, the covers of branching corals, octocorals, encrusting corals and leaf corals were positively correlated with α ¯_transects_, β_transects_ and γ-diversity and were higher in 2000, showing a strong cover decrease from 2005 to 2010. However, the coverage of sponges and tabular corals remained constant between the years 2000 and 2010 (Fig. 4). In general, RDA outcomes demonstrated that a loss of live coral cover (primarily mound, branching, encrusting, and leaf corals as well as octocorals) and a strong increase in fleshy macroalgae (shift transition) (also see Figs. 3A and 3B) and boulder corals reduced all diversity additive components (α ¯, β and γ) due to local extinctions of rare species and unshared species (Fig. 5).

## Discussion

In both geomorphological units, the total species richness dropped significantly over time during the study period. Nevertheless, the total species richness and abundance remained particularly high on the reef terrace, emphasizing the important role of deep geomorphological units and coral cover as a diversity source (Fig. 2). It has recently been suggested that reef geomorphology plays an important role in shaping fish assemblages and β-diversity [4], [45], [71]. Coral assemblages on the reef terrace were more resistant to degradation in comparison to assemblages on the reef slope. In fact, the degradation on the reef terrace occurred later than the general degradation of Quintana Roo’s coral reefs in 2000 [41]. The important decrease in the number of species on the reef terrace from the onset of the study and a lack of substantial differences between subsequent years suggest that the fish assemblage composition on the reef slope had already been affected by a shift transition at the beginning of the study period. Therefore, the total species richness on this geomorphological system was similar across all years.

Interactions among α-, β- and γ-diversity in our study exhibited the expected pattern. It has been suggested, and it is expected, that in a threatened or collapsed system, α-diversity increases at the expense of β-diversity [72]. This pattern was not observed for the reef terrace in terms of total species numbers (Fig. 3). In this case, α-diversity decreased at the same time as β-diversity and γ-diversity. Hewitt et al. [72] found that when a system is threatened, the less vulnerable species become more common. This could explain the moderate decrease in α-diversity, which was not as sharp as the decreases in β- and γ-diversity. In addition, at a certain threshold, α-diversity also decreases and a depauperate, homogeneous community of small, robust species begins to develop. Layou (in [73]), in a paleontological study at a regional scale, found that when extinction was selective for rare taxa, α-diversity steadily increased across all values of extinction percentage, primarily at the expense of among-habitat β-diversity, suggesting that the loss of rare taxa primarily has the effect of increasing similarity between habitats in terms of taxonomic composition. However, this is not what occurred in the Mahahual reef, as demonstrated in this study. In our study, α-diversity exhibited moderate decreases over the studied years on the reef slope, reef terrace and the whole reef as β-diversity and γ-diversity declined (Fig. 4), which suggests that fish assemblages had already crossed a threshold level, at which point the reef entered into a probable regime phase. At this level, all additive diversity components decrease, fish assemblages become more homogeneous, and a compact group of small species becomes common. The most frequent and abundant fish species on the reef are those that contributed most to α-diversity (Table S2) and were apparently moderately affected by the shift transition. Many of these species are small and related to the availability of shelter (e.g., crevices) [39] provided by live or dead coral colonies. Several other rare fish species have a greater dependence on biotic characteristics such as recruitment, mortality, competition, and predation [74], [75], which has enabled them to persist in the system (Fig. 4E). The percentage of rare species (37.1% of singletons and doubletons) on the reef terrace in 2000 when the shift transition was underway was similar to reports from other studies in marine ecosystems [72]. In 2005 after the regime shift, the percentage of rare species was 30.4%. The number of unshared species between the years 2000 and 2005 at the reef level was 39 species (49.4% of total species); that between 2006 and 2010 was 15 species (25.9%). This shows that a group of less sensitive species became more common (Table S2) and that a high number of rare species that declined drastically were sensitive to the regime shift (Table S1). The pattern of rare species decline in ecosystems is in some way polemic because rare species have been implicated as providing insurance and functional resilience against change [72]. It has been demonstrated that the decline of rare species may have profound consequences for ecosystem function by reducing complementary interactions among rare and uncommon species [76]. Less common species can increase the resistance to biological invaders [77] by influencing invasion events, which highlights their important role in ecosystem function [78]. Nevertheless, some studies suggest that the ‘‘insurance’’ provided by rare species in the face of environmental change may be overstated [72]. Our results show that rare species were extremely sensitive to change and they did not become more common during the shift transition, but rather locally disappeared.

Although α, β and γ-diversity decreased at the same time, β-diversity explained the highest proportion of γ-diversity. This implies that even if there is an overall trend of fish assemblage homogenization driven by the loss of rare species, some heterogeneity is maintained. The high β-diversity found in the inter-GU spatial analysis across years means that fish assemblage similarity among nearby habitats is low. This pattern was maintained even after coral reef degradation and regime shift. Dornellas et al. [79] and Hewitt et al. [72] demonstrated that coral and soft sediment communities are markedly different in adjacent similar habitats. This agrees with other studies that found that in ecosystems with a high level of internal heterogeneity, there is an increase in the influence of β-diversity relative to α-diversity [80], particularly for coral reefs. The high contribution of β_transects_ to total fish diversity indicates that habitat and local geomorphological characteristics play a crucial role in fish composition and therefore in ecosystem functioning. The spatial segregation of common species is advantageous to avoid competition for resources. Moreover, when we increased spatial hierarchy and included geomorphological systems in the analysis, β_GU_ represented a higher contribution to total fish diversity as well as, to a lesser extent, β_transects_. This is interesting because, although fish communities tended to be homogeneous between geomorphological units, there was an additional heterogeneity in fish communities that made β-diversity more important than α-diversity for the reef.

In the additive spatial analysis, β_GU_ being higher than expected by chance suggests that fish species composition was influenced by habitat structure and not by random patterns. This shows the importance of former species and the potential impact of shift transitions on species turnover. A growing number of studies have suggested that coral, reef habitat and rugosity are important for coral reef fish β-diversity [4], [5], [17], [19]. With the reef system undergoing a shift transition, restructuring of the composition [35], [81] and proportion of the benthic communities is represented by fleshy macroalgae as well as boulder corals occupying a greater area, causing decreases in the cover of other groups such as mound corals (*i.e. Montastraea* complex), which results in reduced structural complexity [40]. In the year 2000, when shift transition conditions were already apparent on the reef slope and to a lesser extent on the reef terrace, β-diversity had a strong relationship with massive corals such as *Montastraea annularis*. RDA outcomes evidenced that mound corals had a strong negative correlation (high collinearity) with fleshy macroalgae, suggesting a high *Montastraea* complex decrease through the studied years. In contrast, in the years from 2005 to 2010, β-diversity had a strong negative relationship with boulder corals such as *P. astreoides*, which was mainly observed on the reef slope and terrace in 2010 (Fig. 4). This makes it clear that when the abundance of boulder corals increased following the shift transition in the years 2005–2010, β-diversity dropped. The boulder species such as *P. astreoides, Siderastrea* spp. and *Diploria* spp. generate a lower complexity than the *Montastraea* complex because they have a smaller diameter and height [44]. *P. astreoides* and *Diploria* spp. are among the first corals to colonize disturbed reef surfaces, and they have been reported to occur in greater abundance in areas closest to disturbances, which indicates a unique capacity to survive human influence [82], [83]. In addition, the exposed skeletal surfaces of diseased colonies of *M. annularis* have provided substrates that support the recruitment of corals of the genus *Porites* but not *Montastraea* [84].

Other groups of scleractinian corals that enhanced all additive diversity components were branching corals and octocorals. Because they increase topographic complexity and refuge availability, these corals are widely recognized as a critical functional group in coral reefs [85], [86]. They also provide shelter to fish against water movement [87] as well as suitable microhabitats for small predators to use to ambush their prey [88]. Similarly, they provide a variety of physical niches for specific population requirements [86]. Currently, it is known that gorgonians can provide additional or replacement habitat for degrading stony corals [89].

The results of this study have important implications for coral reef ecology and conservation. In ecological terms, our results suggest that a group of common small species slightly decreased and affected fish α-diversity in the reef across the studied years; rare species did not respond well to reef change, diminishing fish β-diversity. Despite this decrease and reef degradation, fish heterogeneity was maintained. In conservation terms, this means that coral reefs appear to maintain some resistance to changes, avoiding some vulnerability to global change. Fish heterogeneity thus possesses some expected ecosystem resilience, and that resilience needs to be managed and conserved cautiously. If the observed patterns of species loss are similar in different reefs under regime shifts in the Caribbean, it is estimated that approximately 22% of rare species would be lost. The capacity to predict diversity patterns is important for the identification of coastal management practices and coral reef conservation areas. In our case, deeper geomorphological areas and mound corals, octocorals, branching corals and other morphofunctional coral groups were identified as crucial sources of diversity and species turnover. This underlines the importance of the effect of habitat homogenization by fleshy macroalgae and boulder corals on biodiversity patterns. The additive partitioning analysis was a useful indicator of diversity heterogeneity in a coral reef system in regime shift.

## Supporting Information

Figure S1 Individual-based rarefaction curves for the reef terrace (A), reef slope (B), and the whole reef (C) in the years 2000, 2005, 2006, 2007, 2008, and 2010.
(PDF)

Figure S2 Species rarity based on abundance and incidence by geomorphological units and whole reef. Numbers of singletons and doubletons (A). Numbers of unique and duplicate species (B).
(TIF)

Table S1 Shared and unshared fish species from reef terrace, reef slope, and reef between years. Singletons = S, doubletons = D, unique species = U, duplicate species = DU, and invasive specie = ***.
(PDF)

Table S2 The top 10 species that contribute more strongly to the dissimilarity (SIMPER). Average dissimilarity = AD.
(PDF)
