# Supplementary material for: Additive Diversity Partitioning of Fish in a Caribbean Coral Reef Undergoing Shift Transition
Source: PLoS One. 2013 Jun 11;8(6):e65665. doi: 10.1371/journal.pone.0065665 (PMC3679153; doi:10.1371/journal.pone.0065665)
Supplement: Figure S1 — Individual-based rarefaction curves for the reef terrace (A), reef slope (B), and the whole reef (C) in the years 2000, 2005, 2006, 2007, 2008, and 2010. (PDF) [file pone.0065665.s001.pdf]

Figure S1. Individual-based rarefaction curves for the reef terrace (A), reef slope (B), and the whole reef (C) in the years 2000, 2005, 2006, 2007, 2008, and 2010.

A

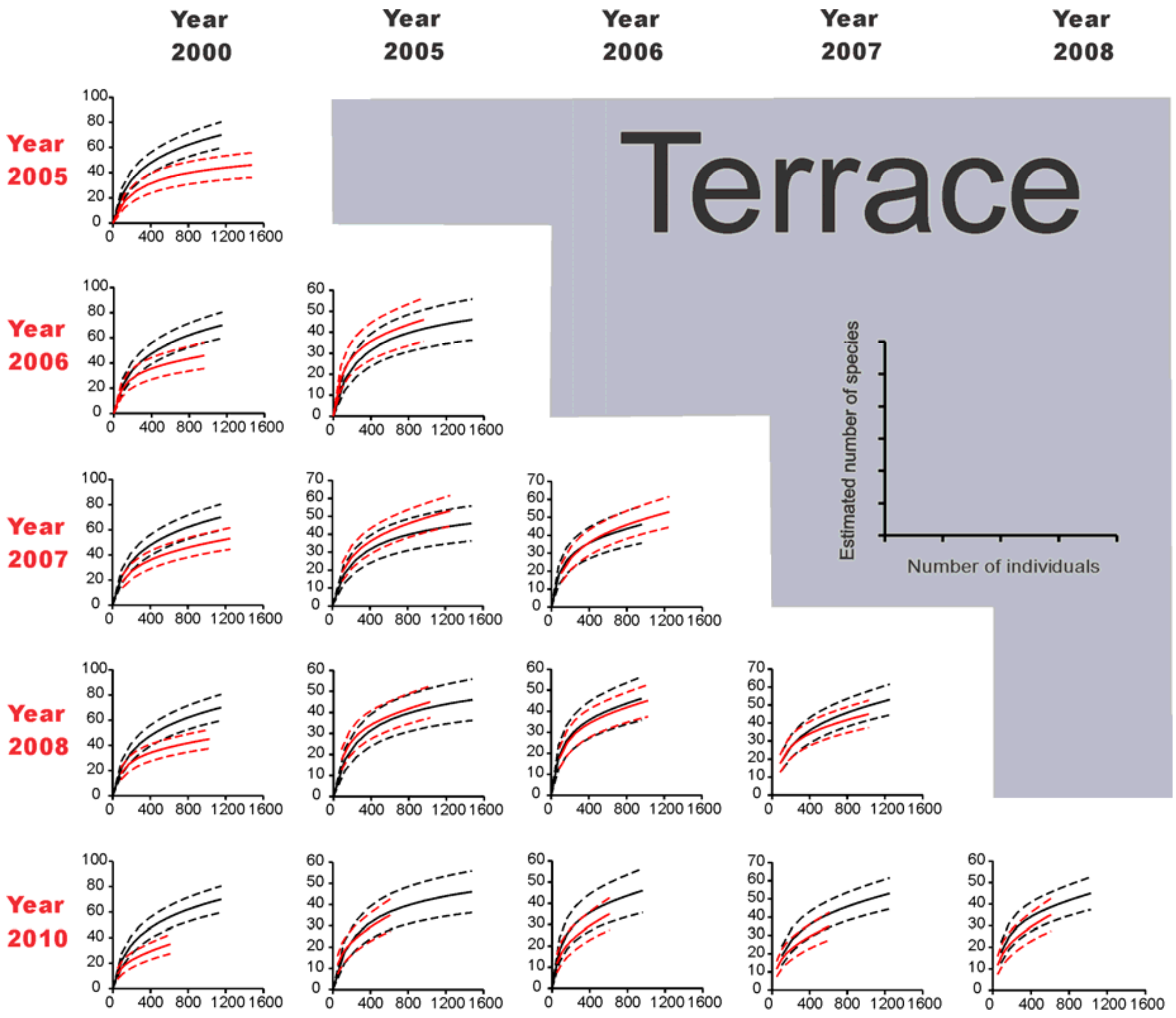

B

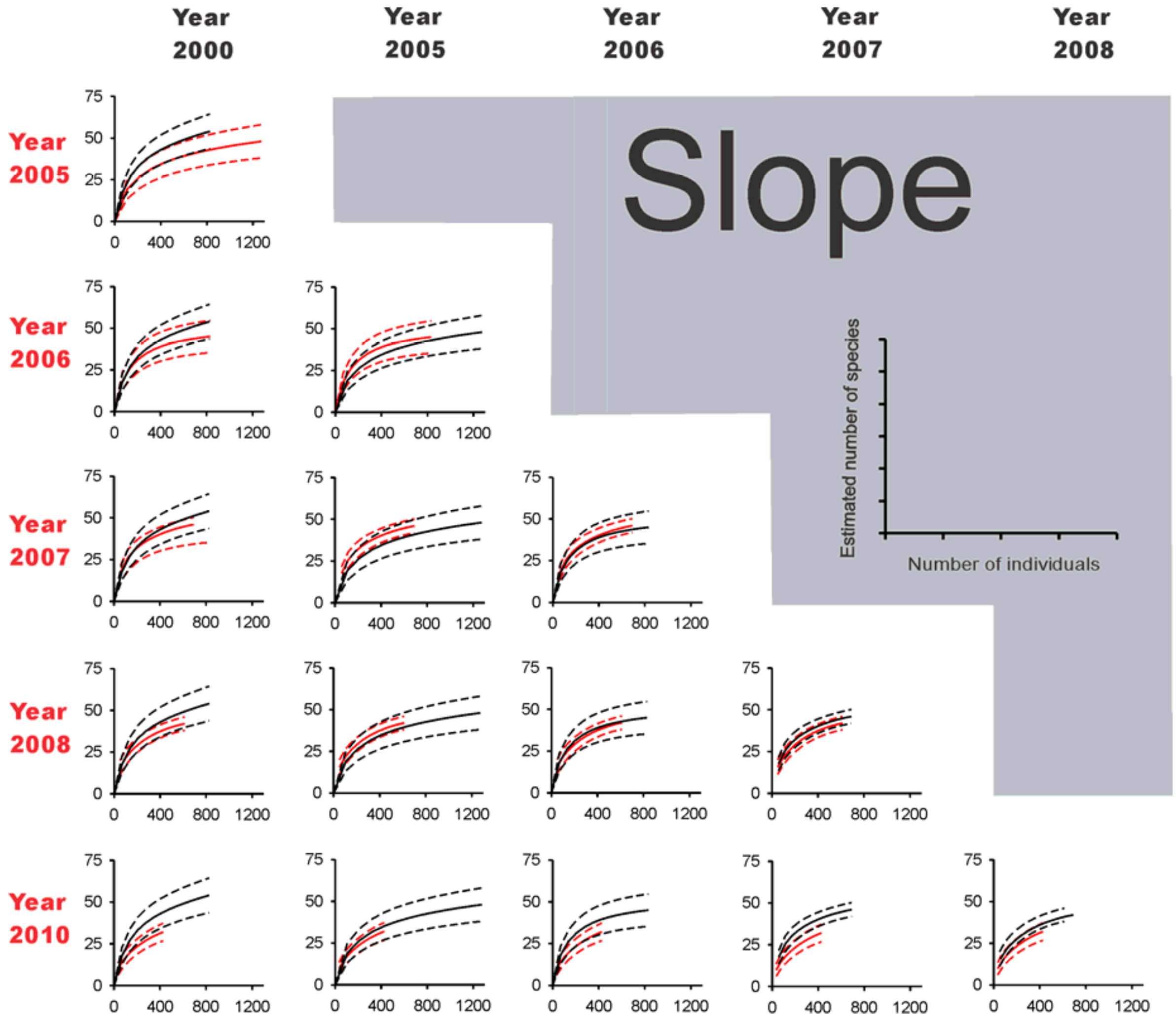
Year 2000
Year 2005
Year 2006
Year 2007
Year 2008
Year 2005
Year 2006
Year 2007
Year 2008
Year 2010
Slope
Estimated number of species
Number of individuals
75
50
25
0
0
400
800
1200

C

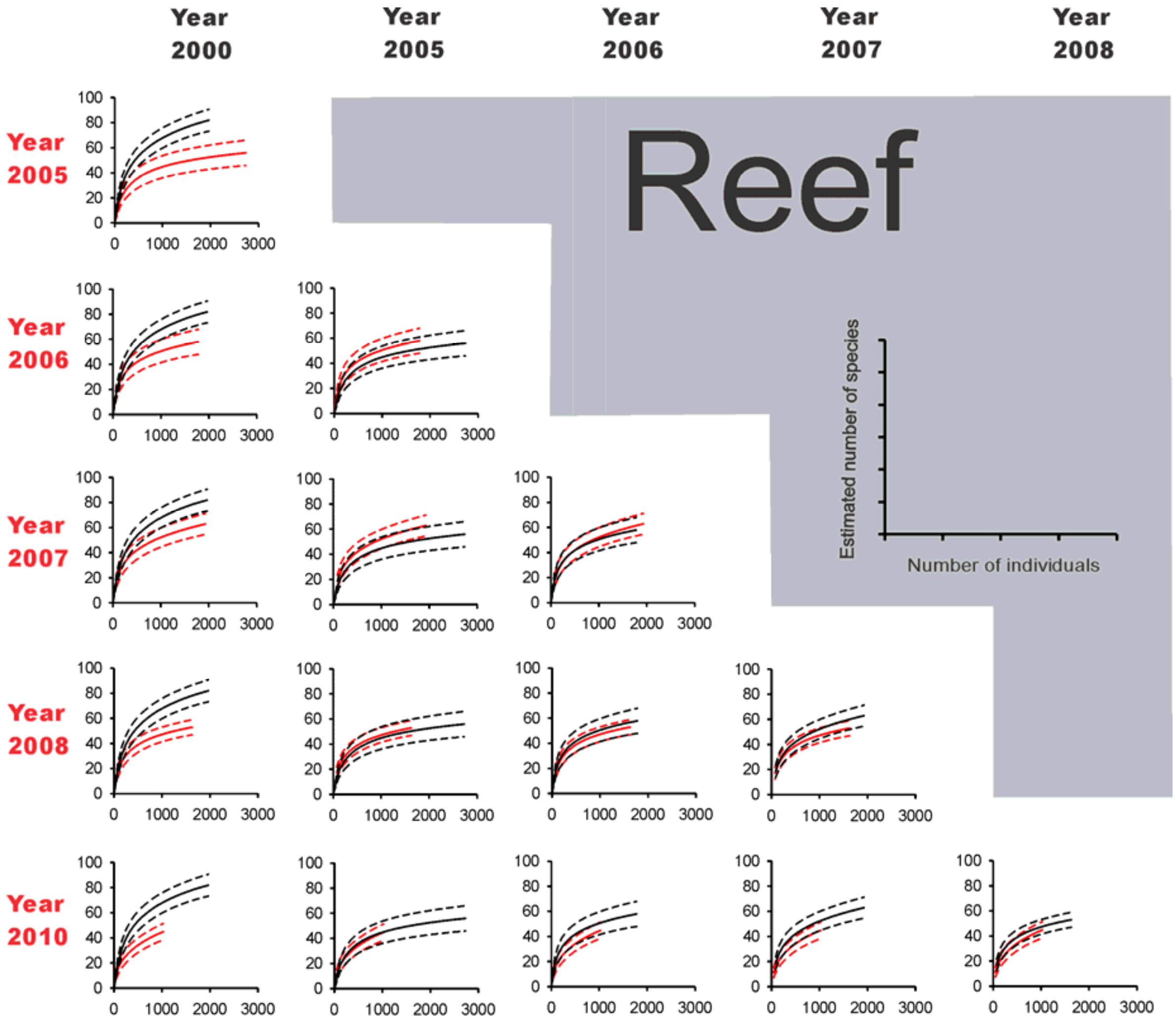
Year 2000
Year 2005
Year 2006
Year 2007
Year 2008
Year 2005
Year 2006
Year 2007
Year 2008
Year 2010
Reef
Estimated number of species
Number of individuals
0
20
40
60
80
100
0
1000
2000
3000
