# Supplementary figures and images for: Additive Diversity Partitioning of Fish in a Caribbean Coral Reef Undergoing Shift Transition
Source: PLoS One. 2013 Jun 11;8(6):e65665. doi: 10.1371/journal.pone.0065665 (PMC3679153; doi:10.1371/journal.pone.0065665)

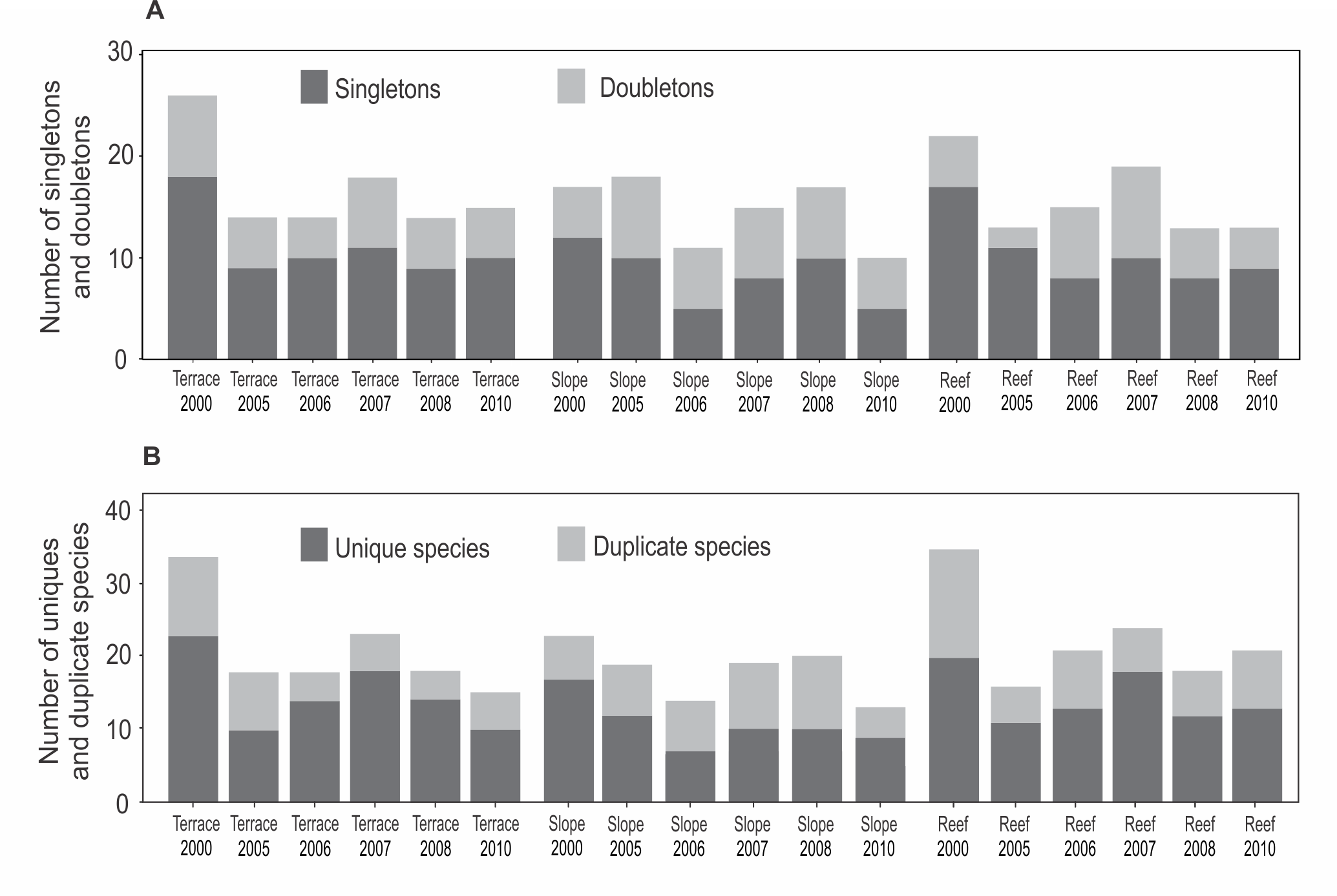

Supplement: Figure S2 — Species rarity based on abundance and incidence by geomorphological units and whole reef. Numbers of singletons and doubletons (A). Numbers of unique and duplicate species (B). (TIF) [file pone.0065665.s002.tif]
