# Supplementary material for: Additive Diversity Partitioning of Fish in a Caribbean Coral Reef Undergoing Shift Transition
Source: PLoS One. 2013 Jun 11;8(6):e65665. doi: 10.1371/journal.pone.0065665 (PMC3679153; doi:10.1371/journal.pone.0065665)
Supplement: Table S1 — Shared and unshared fish species from reef terrace, reef slope, and reef between years. Singletons = S, doubletons = D, unique species = U, duplicate species = DU, and invasive specie = ***. (PDF) [file pone.0065665.s003.pdf]

Table S1. Shared and unshared fish species from reef terrace, reef slope, and reef between years. Singletons = S, dobletons = D, unique species =U, duplicate species = DU, and invasive specie =***.

**TERRACE**

| Unshared species Year 2000 | Unshared species Year 2005 | Unshared species Year 2006 | |
|---|---|---|---|
| *Acanthostracion quadricornis* (S) (U) | *Amblycirrhitus pinos* (S) (U) | *Epinephelus striatus* (S) (U) | |
| *Aetobatus narinari* (S) (U) | *Aulostomus maculatus* (S) (U) | *Halichoeres bivittatus* (U) | |
| *Anisotremus surinamensis* (DU) | *Haemulon chrysargyreum* (S) (U) | *Hypoplectrus indigo* (S) (U) | |
| *Calamus bajonado* (S) (U) | | | |
| *Chromis scotti* (DU) | | | |
| *Elacatinus evelynae* (DU) | | | |
| *Elacatinus oceanops* (DU) | | | |
| *Haemulon macrostomum* (S) (U) | | | |
| *Hypoplectrus unicolor* (S) (U) | | | |
| *Lactophrys bicaudalis* (S) (U) | | | |
| *Neoniphon marianus* (D) (DU) | | | |
| *Odontoscion dentex* (S) (U) | | | |
| *Prognathodes aculeatus* (S) (U) | | | |
| *Sargocentron coruscum* (D) (U) | | | |
| *Stegastes leucostictus* | | | |
| **Unshared species Year 2007** | **Unshared species Year 2008** | **Unshared species Year 2010** | **Shared species in the 6 years** |
| *Diodon holocanthus* (S) (U) | *Haemulon album* (S) (U) | *Balistes vetula* (D) (DU) | 16 |
| *Holocentrus rufus* (S) (U) | *Halichoeres radiatus* (S) (U) | *Diodon hystrix* (S) (U) | |
| *Hypoplectrus nigricans* (S) (U) | *Inermia vittata* | *Pterois volitans* (DU)*** | |
| *Lachnolaimus maximus* (D) (U) | *Kyphosus sectator* | | |
| | *Mycteroperca bonaci* (S) (U) | | |

**SLOPE**

| Unshared species Year 2000 | Unshared species Year 2005 | Unshared species Year 2006 |
|---|---|---|
| *Aetobatus narinari* (S) (U) | *Kiphosus sectator* (D) (DU) | |
| *Amblycirrhitus pinos* (S) (U) | *Lutjanus griseus* (S) (U) | |
| *Apogon maculatus* (S) (U) | | |
| *Caranx hippos* (S) (U) | | |
| *Epinephelus adscensionis* (S) (U) | | |
| *Haemulon chrysargyreum* (S) (U) | | |

| | | | |
|---|---|---|---|
| *Haemulon parra* | | | |
| *Halichoeres pictus* | | | |
| *Halichoeres poeyi* (DU) | | | |
| *Hypoplectrus unicolor* (S) (U) | | | |
| *Lutjanus synagris* (S) (U) | | | |
| *Mulloidichthys martinicus* (DU) | | | |

| Unshared species Year 2007 | Unshared species Year 2008 | Unshared species Year 2010 | Shared species in the 6 years |
|---|---|---|---|
| *Chromis multilineata* (S) (U) | *Lutjanus jocu* (D) (DU) | *Acanthurus chirurgus* | *16* |
| *Epinephelus striatus* (DU) | *Opistognathus aurifrons* (S) (U) | *Anisotremus virginicus* (U) | |
| | | *Balistes capriscus* (S) (U) | |
| | | *Haemulon striatum* (D) (U) | |

**REEF**

| Unshared species Year 2000 | Unshared species Year 2005 | Unshared species Year 2006 | |
|---|---|---|---|
| *Acanthostracion quadricornis* (S) (U) | | *Hypoplectrus indigo* (S) (U) | |
| *Aetobatus narinari* (D) (DU) | | | |
| *Anisotremus surinamensis* (DU) | | | |
| *Apogon maculatus* (S) (U) | | | |
| *Calamus bajonado* (S) (U) | | | |
| *Caranx hippos* (S) (U) | | | |
| *Chromis scotti* (DU) | | | |
| *Elacatinus evelynae* (DU) | | | |
| *Elacatinus oceanops* (DU) | | | |
| *Epinephelus adscensionis* (S) (U) | | | |
| *Haemulon macrostomum* (S) (U) | | | |
| *Halichoeres pictus* | | | |
| *Halichoeres poeyi* (DU) | | | |
| *Hypoplectrus unicolor* (D) (DU) | | | |
| *Lutjanus synagris* (S) (U) | | | |
| *Neoniphon marianus* (D) (DU) | | | |
| *Odontoscion dentex* (S) (U) | | | |
| *Prognathodes aculeatus* (S) (U) | | | |

| Unshared species Year 2007 | Unshared species Year 2008 | Unshared species Year 2010 | Shared species in the 6 years |
|---|---|---|---|
| *Diodon holocanthus* (S) (U) | *Haemulon album* (S) (U) | *Balistes capriscus* (S) (U) | *26* |
| *Hypoplectrus nigricans* (S) (U) | *Inermia vittata* | *Balistes vetula* (U) | |

| | | |
|---|---|---|
| *Lachnolaimus maximus* (U) | *Mycteroperca bonaci* (S) (U) | *Diodon hystrix* (S) (U) |
| | *Opistognathus aurifrons* (S) (U) | *Pterois volitans* *** |
