# Supplementary material for: Additive Diversity Partitioning of Fish in a Caribbean Coral Reef Undergoing Shift Transition
Source: PLoS One. 2013 Jun 11;8(6):e65665. doi: 10.1371/journal.pone.0065665 (PMC3679153; doi:10.1371/journal.pone.0065665)
Supplement: Table S2 — The top 10 species that contribute more strongly to the dissimilarity (SIMPER). Average dissimilarity = AD. (PDF) [file pone.0065665.s004.pdf]

Table S2. The top 10 species that contribute more strongly to the dissimilarity (SIMPER). Average dissimilarity = AD.

	SIMPER Contribution (%)		SIMPER Contribution (%)		SIMPER Contribution (%)
**Terrace 2000-2005 (AD:72.2)**		**Terrace 2000-2006 (AD:64.7)**		**Terrace 2000-2007 (AD:80.1)**	
Clepticus parrae	14.95	*Clepticus parrae*	14.51	*Thalassoma bifasciatum*	19.96
Chromis cyanea	12.2	*Chromis cyanea*	10.64	*Stegastes partitus*	13.39
Stegastes partitus	12.15	*Stegastes partitus*	5.78	*Chromis cyanea*	11.49
Thalassoma bifasciatum	5.93	*Gramma loreto*	5.34	*Scarus iseri*	4.44
Acanthurus bahianus	5.62	*Thalassoma bifasciatum*	3.96	*Stegastes adustus*	4.22
Halichoeres garnoti	4.33	*Sparisoma aurofrenatum*	3.89	*Halichoeres garnoti*	4.2
Scarus iseri	3.85	*Lutjanus griseus*	3.69	*Sparisoma aurofrenatum*	3.94
Gramma loreto	3.65	*Scarus iseri*	3.29	*Acanthurus bahianus*	3.47
Sparisoma aurofrenatum	3.15	*Stegastes planifrons*	3.07	*Gramma loreto*	3.11
Stegastes planifrons	2.78	*Acanthurus coeruleus*	2.58	*Acanthurus coeruleus*	2.6
**Terrace 2000-2008 (AD:61.3)**		**Terrace 2000-2010 (AD:75.2)**		**Terrace 2005-2006 (AD:62.8)**	
Thalassoma bifasciatum	17.62	*Thalassoma bifasciatum*	20.68	*Clepticus parrae*	18.51
Chromis cyanea	13.67	*Chromis cyanea*	16.42	*Chromis cyanea*	15.54
Stegastes partitus	10.29	*Stegastes partitus*	10.3	*Stegastes partitus*	11.06
Scarus iseri	5.13	*Scarus iseri*	4.37	*Thalassoma bifasciatum*	6.86
Halichoeres garnoti	4.11	*Halichoeres garnoti*	4.37	*Acanthurus bahianus*	6.59
Gramma loreto	3.51	*Sparisoma aurofrenatum*	4.05	*Halichoeres garnoti*	4.49
Sparisoma aurofrenatum	3.42	*Acanthurus coeruleus*	3.08	*Scarus iseri*	3.19
Stegastes adustus	3.38	*Gramma loreto*	3.08	*Stegastes planifrons*	2.98
Clepticus parrae	3.08	*Stegastes adustus*	2.45	*Acanthurus coeruleus*	2.46
Abudefduf saxatilis	2.33	*Clepticus parrae*	2.32	*Sparisoma aurofrenatum*	2.43
**Terrace 2005-2007 (AD:61.8)**		**Terrace 2005-2008 (AD:56)**		**Terrace 2005-2010 (AD:72)**	
Clepticus parrae	20.03	*Chromis cyanea*	18.17	*Chromis cyanea*	17
Chromis cyanea	14.23	*Clepticus parrae*	14.03	*Stegastes partitus*	15.11
Stegastes partitus	11.91	*Stegastes partitus*	11.04	*Clepticus parrae*	11.24
Thalassoma bifasciatum	8.1	*Thalassoma bifasciatum*	7.58	*Thalassoma bifasciatum*	6.94
Acanthurus bahianus	5.84	*Acanthurus bahianus*	6.98	*Acanthurus bahianus*	6.27
Halichoeres garnoti	4.5	*Halichoeres garnoti*	5.48	*Halichoeres garnoti*	5.73
Scarus iseri	3.73	*Scarus iseri*	3.48	*Sparisoma aurofrenatum*	4.09
Stegastes adustus	2.9	*Stegastes planifrons*	3.29	*Scarus iseri*	3.41
Stegastes planifrons	2.83	*Sparisoma aurofrenatum*	2.84	*Acanthurus coeruleus*	3.19
Sparisoma aurofrenatum	2.46	*Inermia vittata*	2.64	*Stegastes adustus*	2.58

Terrace 2006-2007 (AD:61.9)

Clepticus parrae	16.82
Stegastes partitus	12.51
Chromis cyanea	10.69
Thalassoma bifasciatum	8.89
Stegastes planifrons	3.72
Scarus iseri	3.52
Stegastes adustus	3.34
Halichoeres garnoti	2.73
Sparisoma aurofrenatum	2.53
Acanthurus coeruleus	2.35

Terrace 2006-2008 (AD:58.4)

Chromis cyanea	16.73
Clepticus parrae	13.5
Stegastes partitus	11.93
Thalassoma bifasciatum	7.67
Stegastes planifrons	3.76
Inermia vittata	2.97
Acanthurus coeruleus	2.84
Sparisoma aurofrenatum	2.83
Acanthurus bahianus	2.55
Stegastes fuscus	2.44

Terrace 2006-2010 (AD:69.7)

Chromis cyanea	16.67
Clepticus parrae	16.07
Stegastes partitus	7.35
Acanthurus coeruleus	4.68
Sparisoma aurofrenatum	4.45
Thalassoma bifasciatum	4.27
Stegastes planifrons	3.49
Stegastes adustus	3.16
Stegastes fuscus	2.67
Acanthurus bahianus	2.5

Terrace 2007-2008 (AD:58.4)

Chromis cyanea	16.57
Clepticus parrae	16.27
Stegastes partitus	12.19
Thalassoma bifasciatum	8.46
Stegastes planifrons	3.96
Scarus iseri	3.53
Stegastes adustus	3.4
Halichoeres garnoti	2.7
Inermia vittata	2.62
Acanthurus bahianus	2.03

Terrace 2007-2008 (AD:72.6)

Clepticus parrae	16.36
Stegastes partitus	15.39
Chromis cyanea	14.13
Thalassoma bifasciatum	9.36
Scarus iseri	3.61
Stegastes adustus	3.19
Acanthurus coeruleus	2.89
Halichoeres garnoti	2.89
Stegastes planifrons	2.86
Sparisoma aurofrenatum	2.21

Terrace 2008-2010 (AD:73)

Chromis cyanea	22.72
Thalassoma bifasciatum	11.44
Stegastes partitus	7.98
Sparisoma aurofrenatum	4.91
Acanthurus coeruleus	4.67
Scarus iseri	4.3
Stegastes adustus	4.08
Halichoeres garnoti	3.11
Scarus taeniopterus	2.69
Acanthurus bahianus	2.46

Slope 2000-2005 (AD: 70)

Thalassoma bifasciatum	19.47
Chromis cyanea	13.22
Stegastes partitus	12.13
Scarus iseri	6.64
Clepticus parrae	4.57
Halichoeres garnoti	3.26
Stegastes planifrons	3.02
Sparisoma viride	2.93
Abudefduf saxatilis	2.65
Sparisoma aurofrenatum	2.65

Slope 2000-2006 (AD:67.3)

Chromis cyanea	13.21
Clepticus parrae	8.66
Stegastes partitus	8.64
Scarus iseri	7.56
Thalassoma bifasciatum	6.17
Stegastes planifrons	4.77
Sparisoma viride	3.86
Stegastes leucostictus	3.73
Acanthurus coeruleus	3.56
Sparisoma aurofrenatum	2.85

Slope 2000-2007 (AD: 70.5)

Chromis cyanea	13.44
Thalassoma bifasciatum	8.71
Clepticus parrae	8.17
Scarus iseri	6.77
Stegastes partitus	6.76
Stegastes adustus	4.2
Sparisoma viride	4.12
Sparisoma aurofrenatum	3.11
Melichthys niger	2.82
Halichoeres garnoti	2.71

Slope 2000-2008 (AD: 72.3)

Chromis cyanea	14.86
Stegastes partitus	12.66
Thalassoma bifasciatum	7.71

Slope 2000-2010 (AD: 77.9)

Chromis cyanea	15.37
Scarus iseri	7.19
Stegastes adustus	6.33

Slope 2005-2006 (AD: 63.3)

Thalassoma bifasciatum	19.63
Stegastes partitus	12.67
Chromis cyanea	11.23

Scarus iseri	6.39	*Clepticus parrae*	6	*Clepticus parrae*	6.55
Clepticus parrae	5.75	*Acanthurus bahianus*	5.87	*Scarus iseri*	5.47
Acanthurus bahianus	5.42	*Sparisoma viride*	5.29	*Stegastes planifrons*	5.23
Sparisoma viride	4.45	*Sparisoma aurofrenatum*	4.12	*Halichoeres garnoti*	3.77
Melichthys niger	2.93	*Thalassoma bifasciatum*	4.04	*Sparisoma aurofrenatum*	3.05
Sparisoma aurofrenatum	2.57	*Acanthurus coeruleus*	3.34	*Acanthurus coeruleus*	2.77
Halichoeres garnoti	2.11	*Abudefduf saxatilis*	3.2	*Stegastes leucostictus*	2.67

**Slope 2005-2007 (AD: 61.9)**		**Slope 2005-2008 (AD: 61.2)**		**Slope 2005-2010 (AD: 81.8)**	
Thalassoma bifasciatum	17.77	*Thalassoma bifasciatum*	22.23	*Thalassoma bifasciatum*	20.91
Stegastes partitus	11.51	*Chromis cyanea*	12.69	*Stegastes partitus*	14.41
Chromis cyanea	11.48	*Stegastes partitus*	10.92	*Chromis cyanea*	10.6
Scarus iseri	5.2	*Scarus iseri*	5.38	*Scarus iseri*	4.43
Clepticus parrae	4.89	*Acanthurus bahianus*	4.08	*Halichoeres garnoti*	4.36
Halichoeres garnoti	4.12	*Halichoeres garnoti*	3.57	*Stegastes adustus*	4.35
Stegastes adustus	3.64	*Sparisoma aurofrenatum*	3.49	*Sparisoma aurofrenatum*	4.29
Sparisoma aurofrenatum	3.52	*Stegastes planifrons*	3.47	*Acanthurus bahianus*	3.67
Stegastes planifrons	3.29	*Gramma loreto*	2.88	*Stegastes planifrons*	2.97
Gramma loreto	2.69	*Abudefduf saxatilis*	2.49	*Acanthurus coeruleus*	2.64

**Slope 2006-2007 (AD: 65.9)**		**Slope 2006-2008 (AD: 66.1)**		**Slope 2006-2010 (AD: 79.7)**	
Stegastes partitus	10.72	*Stegastes partitus*	14.84	*Stegastes partitus*	9.42
Clepticus parrae	10.34	*Thalassoma bifasciatum*	9.89	*Chromis cyanea*	8.64
Thalassoma bifasciatum	9.73	*Chromis cyanea*	8.56	*Clepticus parrae*	7.79
Chromis cyanea	7.31	*Clepticus parrae*	8.33	*Thalassoma bifasciatum*	6.47
Scarus iseri	5.13	*Stegastes planifrons*	5.5	*Stegastes adustus*	6.11
Stegastes planifrons	5.11	*Acanthurus bahianus*	4.99	*Stegastes planifrons*	5.29
Stegastes adustus	4.43	*Scarus iseri*	4.72	*Acanthurus bahianus*	5.01
Acanthurus coeruleus	3.99	*Acanthurus coeruleus*	3.85	*Sparisoma aurofrenatum*	4.77
Sparisoma aurofrenatum	3.7	*Sparisoma aurofrenatum*	3.37	*Acanthurus coeruleus*	4.76
Halichoeres garnoti	3.51	*Stegastes fuscus*	3.08	*Scarus iseri*	4.75

**Slope 2007-2008 (AD: 60.8)**		**Slope 2007-2010 (AD: 73.8)**		**Slope 2008-2010 (AD: 75.4)**	
Stegastes partitus	14.05	*Thalassoma bifasciatum*	11.39	*Stegastes partitus*	17.72
Thalassoma bifasciatum	13.19	*Stegastes partitus*	8.91	*Chromis cyanea*	9.84
Chromis cyanea	7.98	*Chromis cyanea*	8.37	*Thalassoma bifasciatum*	8.86
Clepticus parrae	5.83	*Acanthurus bahianus*	5.89	*Stegastes adustus*	7.76
Acanthurus bahianus	5.75	*Stegastes adustus*	5.86	*Acanthurus bahianus*	6.08
Stegastes adustus	5.52	*Sparisoma aurofrenatum*	5.37	*Halichoeres garnoti*	4.22
Scarus iseri	4.14	*Clepticus parrae*	5.28	*Acanthurus coeruleus*	4.07

Sparisoma aurofrenatum	4.04	*Scarus iseri*	4.64	*Sparisoma viride*	3.91
Halichoeres garnoti	3.43	*Acanthurus coeruleus*	4.39	*Sparisoma aurofrenatum*	3.79
Acanthurus coeruleus	3.07	*Sparisoma viride*	3.57	*Scarus iseri*	3.4

**Reef 2000-2005 (AD: 71.9)**		**Reef 2000-2006 (AD: 67.6)**		**Reef 2000-2007 (AD: 71.9)**	
Chromis cyanea	12.94	*Chromis cyanea*	12.05	*Clepticus parrae*	12.53
Thalassoma bifasciatum	12.34	*Clepticus parrae*	11.99	*Chromis cyanea*	10.94
Stegastes partitus	12.08	*Stegastes partitus*	7.2	*Stegastes partitus*	9.88
Clepticus parrae	10.33	*Scarus iseri*	5.49	*Thalassoma bifasciatum*	8.4
Scarus iseri	5.15	*Thalassoma bifasciatum*	5.1	*Scarus iseri*	5.29
Halichoeres garnoti	3.76	*Stegastes planifrons*	3.94	*Stegastes adustus*	3.39
Acanthurus bahianus	3.52	*Sparisoma aurofrenatum*	3.26	*Sparisoma aurofrenatum*	2.97
Sparisoma aurofrenatum	2.86	*Acanthurus coeruleus*	3.06	*Stegastes planifrons*	2.82
Stegastes planifrons	2.77	*Gramma loreto*	2.83	*Gramma loreto*	2.66
Gramma loreto	2.66	*Sparisoma viride*	2.78	*Sparisoma viride*	2.47

**Reef 2000-2008 (AD: 71.7)**		**Reef 2000-2010 (AD: 76)**		**Reef 2005-2006 (AD: 64.7)**	
Chromis cyanea	14.42	*Chromis cyanea*	15.14	*Chromis cyanea*	14.04
Stegastes partitus	13	*Clepticus parrae*	8.87	*Thalassoma bifasciatum*	13.15
Clepticus parrae	8.2	*Scarus iseri*	5.28	*Clepticus parrae*	12.76
Thalassoma bifasciatum	6.86	*Stegastes adustus*	4.5	*Stegastes partitus*	11.57
Scarus iseri	4.83	*Sparisoma aurofrenatum*	3.99	*Scarus iseri*	4.3
Acanthurus bahianus	3.76	*Acanthurus bahianus*	3.91	*Stegastes planifrons*	4.02
Stegastes planifrons	3	*Sparisoma viride*	3.65	*Halichoeres garnoti*	4.01
Gramma loreto	2.8	*Acanthurus coeruleus*	3.45	*Acanthurus bahianus*	3.94
Sparisoma aurofrenatum	2.76	*Thalassoma bifasciatum*	3.29	*Acanthurus coeruleus*	2.69
Sparisoma viride	2.69	*Stegastes partitus*	3.24	*Sparisoma aurofrenatum*	2.68

**Reef 2005-2007 (AD: 63)**		**Reef 2005-2008 (AD: 60)**		**Reef 2005-2010 (AD: 78.3)**	
Chromis cyanea	13.88	*Chromis cyanea*	17.53	*Chromis cyanea*	15.14
Clepticus parrae	13.32	*Thalassoma bifasciatum*	14.07	*Stegastes partitus*	14.57
Thalassoma bifasciatum	12.06	*Stegastes partitus*	11.08	*Thalassoma bifasciatum*	13.87
Stegastes partitus	11.9	*Clepticus parrae*	8.04	*Clepticus parrae*	6.06
Scarus iseri	4.33	*Acanthurus bahianus*	5.33	*Halichoeres garnoti*	4.95
Halichoeres garnoti	4.27	*Halichoeres garnoti*	4.39	*Acanthurus bahianus*	4.68
Acanthurus bahianus	3.84	*Scarus iseri*	4.32	*Sparisoma aurofrenatum*	4.11
Stegastes adustus	3.21	*Stegastes planifrons*	3.14	*Scarus iseri*	3.88
Sparisoma aurofrenatum	2.93	*Sparisoma aurofrenatum*	3.12	*Stegastes adustus*	3.46
Stegastes planifrons	2.85	*Gramma loreto*	2.12	*Acanthurus coeruleus*	2.89

Reef 2006-2007 (AD: 65.5)		Reef 2006-2008 (AD: 64.6)		Reef 2006-2010 (AD:76.8)	
Clepticus parrae	14.8	*Chromis cyanea*	15.15	*Chromis cyanea*	14.48
Stegastes partitus	11.34	*Stegastes partitus*	12.98	*Clepticus parrae*	11.56
Chromis cyanea	10.11	*Clepticus parrae*	11.23	*Stegastes partitus*	8.73
Thalassoma bifasciatum	9.1	*Thalassoma bifasciatum*	8.21	*Thalassoma bifasciatum*	5.28
Stegastes planifrons	4.29	*Stegastes planifrons*	4.57	*Acanthurus coeruleus*	4.62
Scarus iseri	4.16	*Acanthurus bahianus*	3.53	*Stegastes adustus*	4.5
Stegastes adustus	3.76	*Scarus iseri*	3.32	*Sparisoma aurofrenatum*	4.47
Acanthurus coeruleus	3.04	*Acanthurus coeruleus*	3.28	*Stegastes planifrons*	4.31
Sparisoma aurofrenatum	3.02	*Sparisoma aurofrenatum*	2.97	*Acanthurus bahianus*	3.53
Halichoeres garnoti	3.02	*Chromis cyanea*	15.15	*Halichoeres garnoti*	2.81

Reef 2007-2008 (AD:63)		Reef 2007-2010 (AD:75.5)		Reef 2008-2010 (AD:73)	
Chromis cyanea	14.85	*Chromis cyanea*	13.72	*Chromis cyanea*	18.68
Stegastes partitus	13.74	*Stegastes partitus*	12.38	*Stegastes partitus*	17.38
Clepticus parrae	11.44	*Clepticus parrae*	10.64	*Thalassoma bifasciatum*	8.08
Thalassoma bifasciatum	9.93	*Thalassoma bifasciatum*	10.12	*Stegastes adustus*	5.04
Stegastes adustus	4.1	*Stegastes adustus*	4.19	*Acanthurus bahianus*	4.39
Acanthurus bahianus	3.61	*Scarus iseri*	3.96	*Acanthurus coeruleus*	3.87
Scarus iseri	3.59	*Sparisoma aurofrenatum*	3.58	*Sparisoma aurofrenatum*	2.86
Halichoeres garnoti	2.87	*Acanthurus coeruleus*	3.54	*Scarus iseri*	2.77
Stegastes planifrons	2.72	*Acanthurus bahianus*	3.47	*Sparisoma viride*	2.71
Sparisoma aurofrenatum	2.69	*Halichoeres garnoti*	3.02	*Scarus taeniopterus*	2.44
